# The Fluoroscopy Paradox: Radiation Exposure, Dose Optimization, and Occupational Risk in Full-Endoscopic and Biportal Spine Surgery—A Narrative Review

**DOI:** 10.3390/jcm15114032

**Published:** 2026-05-22

**Authors:** Dong Hun Kim, Jae-Taek Hong, Jung-Woo Hur

**Affiliations:** 1 Department of Neurosurgery, Bucheon St. Mary’s Hospital, The Catholic University of Korea, Bucheon 14647, Republic of Korea; 2 Department of Neurosurgery, Eunpyeong St. Mary’s Hospital, The Catholic University of Korea, Seoul 03312, Republic of Korea

**Keywords:** endoscopic spine surgery, full-endoscopic spine surgery, unilateral biportal endoscopy, fluoroscopy, radiation exposure, occupational dose, ALARA, navigation, dose optimization

## Abstract

Endoscopic spine surgery (ESS)—including full-endoscopic transforaminal and interlaminar techniques, and unilateral biportal endoscopy (UBE)—offers patients smaller incisions, preserved paraspinal muscle, and faster recovery. Because the working corridor is narrow, intraoperative fluoroscopy plays a larger role than in open or microscopic approaches, making radiation exposure worthy of attention for both patients and surgeons. This narrative review aims to be a practical resource for the endoscopic spine surgeon. We synthesize the available literature on typical radiation doses across the main ESS techniques, compare them with minimally invasive transforaminal lumbar interbody fusion (MIS-TLIF) and open alternatives, review the factors that drive exposure, and walk through the full menu of dose-optimization options—from simple measures such as collimation, pulsed fluoroscopy, and leaded eyewear, through navigation platforms, to robotic guidance. A consistent practical observation is that the simplest, least expensive interventions often deliver the largest dose reductions. Capital-intensive technologies add real value, particularly for endoscopic interbody fusion, and work best alongside rather than in place of these basics. With routine dosimetry and straightforward as-low-as-reasonably-achievable (ALARA) practices, surgeons can continue to build on the already favourable profile of ESS while keeping radiation exposure low. Conclusions are tempered by the largely retrospective and heterogeneous nature of the underlying evidence.

## 1. Introduction

Endoscopic spine surgery has moved, in two decades, from a fringe technique performed by a handful of pioneers to a mainstream modality endorsed by major societies and taught at dedicated international fellowships. The clinical logic is compelling: a working cannula no wider than a ballpoint pen, continuous saline irrigation, magnified visualization of the neural elements, and—for the patient—smaller skin incisions, preserved paraspinal musculature, and earlier mobilization [1,2]. What the marketing rarely foregrounds is the price paid in ionizing radiation.

Unlike open or microscopic surgery, where the anatomy is visible through the wound, the endoscopic surgeon operates through an indirect corridor whose position must be verified radiographically before, during, and often after every critical step. The transforaminal approach depends on repeated biplanar fluoroscopic confirmation during needle placement, serial dilation, foraminoplasty, and working cannula docking. Biportal endoscopy reduces intraoperative fluoroscopy once the working space is established but still relies on C-arm guidance for the initial trajectory and, in fusion variants, for the cage and pedicle-screw placement. Endoscopic lumbar interbody fusion multiplies this dependence further. A practical pattern emerges: techniques that minimize tissue disruption tend to depend more heavily on imaging.

The problem has three intertwined dimensions. First, the patient receives an effective dose that—while typically below thresholds for deterministic harm—is not negligible and accumulates across serial imaging, particularly in patients destined for revision surgery. Reported per-case patient effective doses for transforaminal full-endoscopic lumbar discectomy cluster between 0.6 and 2.1 millisieverts (mSv), with L5–S1 procedures approaching double the dose of cranial levels [3]. Second, the surgeon and operating room team accrue low but chronic occupational exposure, and because spine fluoroscopy is often performed with the tube beneath the table and the operator leaning over the beam path, the scatter dose to the eyes, thyroid, and hands can be disproportionately high. Third, the regulatory and occupational health framework—the as-low-as-reasonably-achievable (ALARA) principle, International Commission on Radiological Protection (ICRP) dose limits, and cataract-risk surveillance—was developed largely with interventional radiology and cardiology in mind and has been only incompletely implemented in spine-surgical practice.

Prior reviews on radiation in spine surgery each leave a specific gap. The systematic review by Srinivasan and colleagues was published in 2014, predating the diffusion of unilateral biportal endoscopy, and does not address full-endoscopic transforaminal versus interlaminar approaches as distinct dose signatures. A systematic review by Arif and colleagues focuses exclusively on surgeon dose in minimally invasive spine surgery and does not examine patient dose or the surgeon-versus-patient trade-offs inherent to cone-beam computed tomography (CBCT)-based navigation. A narrative by Jenkins and colleagues addresses radiation in general spine surgery without ESS-specific analysis. A 2025 multi-specialty review by Helton and colleagues covers surgical fluoroscopy exposure across many disciplines, but treats spine surgery as one aspect rather than as a focus [4]. The present review attempts to fill this gap by integrating the post-2020 UBE and endoscopic-fusion literature, framing patient and surgeon doses as analytically separable problems, and walking through the full intervention hierarchy from zero-cost technique modifications to robotics. We frame the resulting synthesis as a practical conceptual organizer for the endoscopic spine surgeon rather than as a novel analytical model.

We accordingly address four questions. What is the magnitude of intraoperative radiation exposure in contemporary ESS, for both patient and surgical team? How does this compare with MIS-TLIF and conventional open approaches? Which dose-optimization strategies are supported by evidence, and at what cost? And what are the occupational and practical implications for the endoscopic spine surgeon?

## 2. Materials and Methods

This narrative review was prepared in accordance with the Scale for the Assessment of Narrative Review Articles (SANRA) criteria. A structured literature search of PubMed/MEDLINE, Embase, and the Cochrane Library was performed for studies published between January 2010 and April 2026, with selected seminal earlier studies retained where directly relevant to dosimetric methodology [5].

### 2.1. Search Strategy

Search terms combined Medical Subject Headings (e.g., “Diskectomy, Percutaneous,” “Neuroendoscopy,” “Fluoroscopy,” “Radiation Dosage,” “Occupational Exposure,” “Radiation Protection”) with free-text terms for full-endoscopic spine surgery, unilateral biportal endoscopy, MIS-TLIF, dose–area product (DAP), and personal protective equipment. Reference lists of retrieved articles were hand-searched. Grey literature (ICRP, International Atomic Energy Agency [IAEA], national radiation-protection agency publications) was searched directly. Date of last search: 12 April 2026.

### 2.2. Inclusion and Exclusion Criteria

Inclusion criteria: (i) original studies reporting quantitative patient or surgeon dosimetry (DAP, fluoroscopy time, effective dose, or organ-equivalent dose) in endoscopic or fluoroscopy-dependent minimally invasive spine surgery; (ii) systematic reviews, meta-analyses, and narrative reviews relevant to dose optimization in spine surgery; (iii) ICRP and IAEA guidance documents addressing occupational and patient radiation protection in interventional procedures; (iv) studies on personal protective equipment, navigation, or robotic guidance with direct relevance to ESS workflow.

Exclusion criteria: (i) studies confined to thoracic or cervical procedures unless directly relevant to lumbar dose-reduction strategies; (ii) case reports without dosimetric data; (iii) studies reporting only diagnostic radiology dose; (iv) non-English-language publications without English-language abstracts.

### 2.3. Heterogeneity, Selection Bias, and Limitations of the Included Evidence

The included studies are heterogeneous along several dimensions that should temper how the reader interprets the figures we cite. Dosimetric metrics vary across studies (fluoroscopy time only, DAP, effective dose, organ-equivalent dose), making direct numerical comparison difficult. Surgeon experience is rarely standardized and ranges from early learning curve to high-volume practice; the same procedure measured at different points on a single surgeon’s curve can differ by a factor of three or more. Patient body habitus, the level operated, the proportion of revision cases, the choice of pulsed versus continuous fluoroscopy, and the routine versus selective use of intraoperative CBCT each substantially affect reported doses, yet stratification by these variables is uncommon in the source literature. Most published series are single-centre and retrospective, with selection biases that favour established practices and well-equipped institutions; multicentre prospective dosimetry registries do not yet exist in spine surgery. We have therefore presented dose ranges rather than point estimates wherever feasible, and have weighted prospective dosimetric studies (Table 1 and Table 2) more heavily than retrospective comparative work.

This review is not a systematic review or meta-analysis. Quantitative synthesis was not performed, and we make no claim of exhaustive coverage. The intent is a practical, audience-facing organization of the evidence for the working endoscopic spine surgeon.

### 2.4. Use of Generative AI Tools

In accordance with MDPI’s policy on the use of generative artificial intelligence (AI) and AI-assisted technologies in scholarly publication, the authors disclose the following. A large-language-model assistant (Claude Opus 4.6, Anthropic, San Francisco, CA, USA) was used to support the drafting and revision of the full manuscript text, including the organization of sections, the synthesis of literature-derived material into prose, the preparation of tables, and language editing across successive revision rounds. The AI assistant did not perform any of the underlying scientific work: the literature search strategy, the selection and appraisal of included studies, the extraction and interpretation of dosimetric data, the clinical judgements expressed throughout the review, and the conclusions are entirely those of the authors. All AI-generated text was reviewed, verified, edited, and approved by the authors, who take full responsibility for the accuracy, integrity, and scientific content of the work. Following an editorial team query about reference accuracy, every cited reference was re-verified by the authors against the original publications and the corresponding PubMed and DOI records; corrections were applied where AI-assisted drafting had introduced inaccuracies in author lists, page ranges, or DOI suffixes.

## 3. Radiation Dosimetry: Essentials for the Endoscopic Spine Surgeon

Much of the confusion in the ESS literature comes from authors using “fluoroscopy time” and “radiation dose” as if they were interchangeable. They are not, and consistent terminology is essential when interpreting the comparative data presented later in this review.

Fluoroscopy time (seconds) is the easiest metric to record, but a poor proxy for dose. Two operators with identical beam-on time can deliver very different doses depending on continuous versus pulsed mode, collimation, magnification, patient size, and machine settings. Where studies report fluoroscopy time alone, we treat the data as a surrogate for procedural radiographic burden rather than as a direct dose measurement, and we flag this where it applies. Dose–area product (DAP), displayed on every modern C-arm console in Gy·cm^2^, captures both beam intensity and irradiated area and is the standard input for patient-dose calculation. Effective dose (mSv) is a whole-body equivalent that allows comparison across procedures and carries an inherent 20–40% uncertainty. Equivalent dose to an organ (mSv) applies specifically to the eye lens, thyroid, or hand, and matters for the surgeon because occupational limits are set organ by organ. A single-level transforaminal endoscopic lumbar discectomy delivers a patient effective dose roughly equivalent to one to two low-dose chest computed tomography (CT) scans.

Biological effects are of two kinds. Deterministic effects (cataract, skin injury) occur above threshold doses; the eye-lens threshold is now recognized as low as 0.5 Gy, which is why the occupational eye-lens limit was sharply reduced in 2011 [15]. Stochastic effects (cancer) have no threshold and accumulate cumulatively, which is why the ALARA principle applies even to low-dose cases [16,17].

The occupational dose limits that an endoscopic surgeon should know are summarized in Table 2.

Two geometric rules follow from the physics of scattered radiation. First, dose falls with the square of distance from the patient—doubling the distance cuts the surgeon’s dose to one-quarter. Second, scatter peaks on the side of the table where the X-ray tube sits; with the tube under the table, lateral and oblique projections direct the most scatter toward the operator. Convention is to wear one badge at collar level outside the apron and a second at waist level beneath it; ring and eye-lens-specific dosimeters are recommended for high-volume operators [18]. Many reported dose discrepancies trace to inconsistent badge placement, reporting fluoroscopy time alone without DAP, or phantom designs that underestimate real-world exposure.

## 4. Radiation Exposure Across Endoscopic Techniques

Endoscopic spine surgery is not a single procedure. The term covers at least four distinct technical families with characteristic fluoroscopy patterns and dose profiles (Table 3). Throughout this section, we report effective dose (mSv) or DAP-derived doses where studies provide them, and fluoroscopy time only where these are not available—recognizing that fluoroscopy time is an imperfect surrogate for actual radiation exposure.

The full-endoscopic transforaminal approach (FE-TF) is the most fluoroscopy-dependent, because needle placement, serial dilation, foraminoplasty, and cannula docking are all guided by biplanar fluoroscopy. The widely cited prospective series from Iprenburg, Wagner, Godschalx, and Telfeian reported mean patient effective doses of 1.5 mSv at L4–5 and above, rising to 2.1 mSv at L5–S1, with a 3.5-fold reduction in dose between the senior surgeon’s first 100 cases and subsequent practice [3]. Other series report per-case patient effective doses from 0.6 to 4 mSv, with variance driven by experience, level, and foraminoplasty. In a prospective study of percutaneous endoscopic lumbar discectomy (PELD) without additional protective measures, the surgeon’s chest-level dose of 0.108 mSv fell to 0.039 mSv with collimation alone [9].

The full-endoscopic interlaminar approach (FE-IL), used predominantly at L5–S1 where the interlaminar window is widest, requires only 5–15 s of fluoroscopy per case with patient effective doses typically below 1 mSv—a three- to fivefold reduction from FE-TF at the same level.

Unilateral biportal endoscopy (UBE/BESS) approaches the canal through a familiar posterior corridor, with the working space maintained under direct endoscopic visualization after initial localization. Most comparative series report lower fluoroscopy times for UBE than for transforaminal FE—typically 10–25 s per case for single-level decompression [12,13]. Because most of these studies report fluoroscopy time rather than effective dose, the proportional dose reduction cannot be inferred precisely. One rigorous comparison found similar exposure between UBE and interlaminar FE [19], suggesting that the difference is specifically against transforaminal FE rather than FE as a whole. Fluoroscopy rises with contralateral decompression and revision cases.

Endoscopic lumbar interbody fusion—whether full-endoscopic (Endo-TLIF) or biportal (UBE-TLIF)—multiplies the radiation burden because fluoroscopy required for cage sizing and percutaneous pedicle-screw placement is added to that of the decompression itself. Reported fluoroscopy times range from 60 to 180 s, with patient effective doses of 3–8 mSv. Pedicle-screw placement alone commonly accounts for more than half of fluoroscopy cases under conventional C-arm guidance—the domain where the dose argument for navigation and robotics becomes strongest.

Three patterns recur. Dose appears to scale with procedural complexity more than with “endoscopic-ness”—endoscopic fusion delivers more radiation than tubular MIS-TLIF, and the “minimally invasive” label offers no radiological reassurance. The learning curve is the single largest modifiable determinant in published series. L5–S1 emerges as a radiological pressure point across all endoscopic techniques.

## 5. Head-to-Head Comparison: FE, UBE, MIS-TLIF, and Open Surgery

### 5.1. Important Caveats on Cross-Study Comparison

Before presenting the comparisons in this section, the reader should hold in mind several structural limitations of the underlying evidence. Almost all head-to-head data come from single-centre series with substantial methodological heterogeneity, and direct numerical comparisons between studies are unavoidably approximate.

Five sources of heterogeneity dominate. Learning-curve effects mean the same procedure can differ by a factor of three or more between an early-curve and a high-volume operator [3]. Body habitus exerts a large but rarely stratified effect: radiation output scales exponentially with tissue thickness, so a patient cohort with a higher mean body mass index (BMI) will appear to give a procedure a higher “intrinsic” dose. Fluoroscopy settings (continuous vs. pulsed, magnification, low-dose mode, last-image-hold) differ across institutions and are inconsistently reported. Revision-case proportions inflate doses in series that include them. Reported metrics differ across studies—some report only fluoroscopy time, others DAP, others derived effective dose—so quantitative pooling across series is inherently approximate. The figures presented below should accordingly be read as orders of magnitude, not precise benchmarks.

### 5.2. Patient Dose

For lumbar discectomy, the hierarchy reported across studies is reasonably consistent, though the absolute magnitudes vary with study design and operator experience. Open microdiscectomy is associated with patient doses typically below 0.1 mSv. Tubular minimally invasive (MIS) microdiscectomy raises this to 0.2–0.5 mSv. FE-interlaminar discectomy at L5–S1 sits below 1 mSv. FE-transforaminal discectomy sits at the upper end of this range at 1.5–2.1 mSv in experienced hands, with higher figures reported during the learning curve [3,11].

For instrumented fusion, minimally invasive transforaminal lumbar interbody fusion (MIS-TLIF) is associated with reported patient doses of 4–10 mSv per single-level case [5]. Open TLIF or posterior lumbar interbody fusion (PLIF) reports lower values, typically 1–3 mSv, reflecting less fluoroscopy dependence because anatomic landmarks are directly visible. Endoscopic fusion sits in the 3–8 mSv range in available series, with higher values during the learning curve or with freehand percutaneous screws.

The headline pattern across multiple series and two systematic reviews is that fluoroscopy-dependent minimally invasive techniques are typically associated with patient doses approximately 5 to 20 times those of their open counterparts, with figures rising further when intraoperative CBCT is added. The O-arm contributes 1–3 mSv per spin, and multi-spin protocols can push a single-level MIS fusion above 10 mSv [8,10]. These ratios should be interpreted cautiously given the heterogeneity discussed in Section 5.1. Table 4 summarizes the comparison.

### 5.3. Surgeon Dose

The surgeon-dose picture is almost the mirror image of the patient-dose picture, because technologies that reduce patient exposure (navigation, O-arm) generally do so by removing the surgeon from the room during acquisition rather than by reducing the total radiation delivered. Reported surgeon unshielded chest-level doses per case are ordered as follows: open microdiscectomy, <0.01 mSv; tubular MIS, 0.01–0.05 mSv; FE-interlaminar, 0.02–0.08 mSv; FE-transforaminal without collimation, 0.1 mSv in mature practice and up to 0.3 mSv during the learning curve [16]; MIS-TLIF under fluoroscopy, 0.3–0.7 mSv; endoscopic fusion without navigation, comparable to MIS-TLIF.

A surgeon performing 200 FE-TF discectomies annually without collimation would accumulate, by simple extrapolation from single-case figures, roughly 20 mSv of unshielded chest-level dose per year. Such per-case-to-annual extrapolations carry substantial uncertainty because they ignore between-case variability, the protective effect of routinely worn personal protective equipment, and the modulating effect of operator experience. Taken with appropriate caution, the estimate nevertheless suggests that high-volume endoscopic practice can approach the whole-body effective-dose limit under the apron and plausibly exceed the eye-lens limit above it. Cross-specialty data are consistent with spine surgery sitting among the higher-exposure surgical disciplines [4], though direct spine-specific occupational cohort data remain limited. The margin between occupational compliance and non-compliance can be narrow in high-volume endoscopic practice, particularly for the eye lens and hands.

### 5.4. The L5–S1 Pressure Point

At L5–S1, FE-TF delivers roughly three to five times the patient dose of FE-IL and twenty to thirty times that of open microdiscectomy. For uncomplicated L5–S1 herniations amenable to both, selection of the interlaminar approach is among the most effective dose-reduction interventions available, with a magnitude comparable to any equipment- or technique-based intervention.

### 5.5. Three Practical Messages

ESS is not uniformly a high-radiation field. FE-interlaminar and UBE decompression sit in the tubular-MIS range, well below MIS-TLIF or any instrumented fusion—the higher doses cluster around transforaminal approaches and endoscopic fusion. The comparison with open surgery favours open surgery on dose alone; patients undergoing a fluoroscopy-dependent “minimally invasive” procedure typically receive 5–20 times the ionizing radiation of an open counterpart, and this deserves explicit acknowledgement in consent. Dose trade-offs between surgeon and patient are real and usually unspoken: CBCT and navigation shift dose from surgeon to patient, while collimation, pulsing, and technique selection reduce both.

## 6. Patient Dose Versus Surgeon Dose: Two Problems, Not One

**Figure 1:**
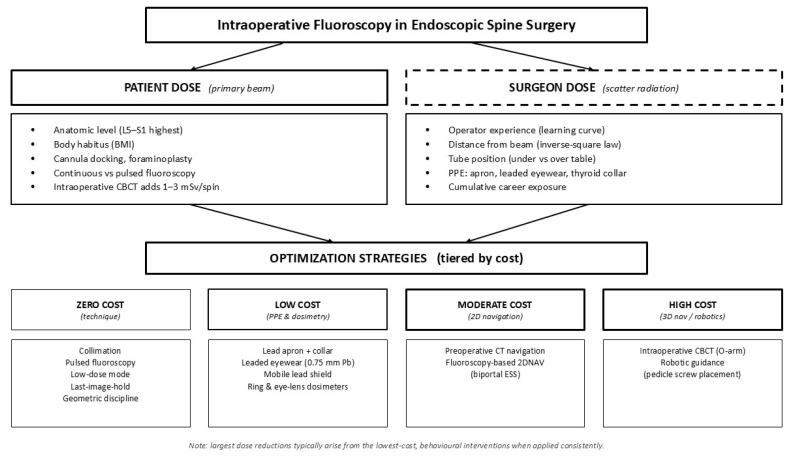
Conceptual framework summarizing patient and surgeon dose drivers in endoscopic spine surgery and the four-tier hierarchy of dose-optimization strategies. ESS, endoscopic spine surgery; BMI, body mass index; CBCT, cone-beam computed tomography; PPE, personal protective equipment; 2DNAV, fluoroscopy-based two-dimensional intraoperative navigation.

Most of the ESS literature treats radiation exposure as a single variable. This obscures an important distinction: patient dose and surgeon dose are governed by different physics, subject to different regulatory frameworks, modified by different interventions, and borne by different people with different stakes in the outcome. Distinguishing them helps explain why certain interventions seem to “reduce radiation” in some studies and appear neutral in others—they may be reducing one party’s dose while increasing the other’s. The conceptual organization is summarized in Figure 1.

The physics differs. Patient dose comes from the primary beam and depends on beam intensity, beam-on time, and the anatomy traversed. Surgeon dose comes from scattered radiation; it depends on distance, scatter direction relative to tube position, and personal shielding. The same intervention can have opposite effects on the two parties. Adding a lead drape reduces the surgeon dose with no effect on the patient dose. Performing an intraoperative CBCT increases the patient dose but usually reduces the surgeon’s dose to zero for that portion of the case. Each intervention has a distinct dose “signature,” and any claim to reduce “radiation exposure” is meaningless without specifying whose dose.

The regulatory frameworks differ. Patient dose is governed by clinical justification and ALARA, with no numeric upper limit—a patient receiving a dose unacceptable as occupational exposure may still be receiving appropriate care. Surgeon dose is governed by enforceable occupational limits: 20 mSv/year whole-body, 20 mSv/year eye lens, 500 mSv/year extremities. A surgeon cannot consent to higher exposure, even if willing, because the framework exists to protect workers from self- or employer-imposed pressure.

The ethical weight differs. Patient dose is a one-time, consented, clinically justified exposure weighed against clinical benefit. Surgeon dose is cumulative, unconsented, structurally imposed, and without per-case clinical benefit to the exposed individual. The historical pattern in medical radiation protection—from early radiologists to interventional cardiologists—has been that the occupational side of the ledger is undercounted until late effects emerge. ESS is early enough in its diffusion curve that the field can avoid repeating that pattern.

### 6.1. The Patient Perspective

Per-case doses in ESS range from well under 1 mSv for FE-IL to 3–8 mSv for endoscopic fusion. For a single procedure, a 2 mSv effective dose—the upper end of a mature-practice FE-TF L5–S1 discectomy—corresponds to an estimated lifetime attributable cancer risk of approximately 1 in 10,000 under the linear-no-threshold model. The calculus changes in specific groups: patients undergoing revision or multi-level surgery accumulate dose across serial episodes; young patients have longer remaining life expectancy over which stochastic effects manifest; patients with hereditary cancer syndromes carry elevated baseline radiosensitivity. None of these perspectives argue against ESS when clinically indicated, but they argue for recording patients’ effective dose in the operative report for future reference.

### 6.2. The Surgeon Perspective

The surgeon’s dose problem is dominated by cumulative career exposure and by organ-specific doses that often exceed predictions from whole-body measurements. The eye lens is the most vulnerable site, with per-case doses of 0.01–0.1 mSv without protective eyewear. Leaded eyewear (0.75 mm Pb equivalent) reduces eye-lens dose by 90% or more and is among the most effective personal protection measures in ESS [20]. The hands receive 0.05–0.3 mSv per case and can approach the 500 mSv annual extremity limit in high-volume practice without cannula-handling discipline. The thyroid is effectively shielded when the collar is worn correctly, but collar displacement during long cases is common.

Radiation is not the only occupational consideration for the endoscopic spine surgeon. The narrow working corridor, indirect endoscopic visualization, prolonged standing, sustained cervical flexion, and static upper-limb postures characteristic of ESS together impose ergonomic strain that has been associated with musculoskeletal complaints across spine surgical disciplines. Ergonomics and radiation interact in practice: a surgeon who leans closer to the operative field to compensate for cumbersome shielding or a suboptimal C-arm geometry simultaneously increases scatter dose. The occupational profile of ESS is therefore best understood as multidimensional rather than radiation-only—a framing we return to in Section 8.5 in the context of emerging wearable monitoring technologies.

The career-dose question remains under-addressed. Epidemiological studies of interventional cardiologists and radiologists have documented elevated rates of left-sided brain tumours, cataracts, and skin changes associated with occupational fluoroscopy [21,22]. The extent to which these findings extrapolate to endoscopic spine surgeons is not yet established. The underlying physics—scatter geometry, beam orientation, cumulative case volumes—suggests that similar risks are biologically plausible, but the published spine literature lacks the large, prospective, multicentre occupational cohorts that would be required to confirm or quantify this directly. Until such data exist, extrapolation from interventional radiology and cardiology should be treated as a hypothesis warranting prudent precaution, not as established causation in spine surgery. Prospective career-dose cohort studies in ESS therefore represent a substantive research priority.

## 7. Determinants of Exposure

Six variables explain most of the between-surgeon and between-institution variance in reported ESS doses, listed here in approximate order of effect size.

Operator experience (the learning curve) is the single largest modifiable determinant. Iprenburg and Telfeian reported a 3.5-fold reduction in patient dose between a senior surgeon’s first 100 cases and subsequent practice [3]. The learning curve is approach-specific—a surgeon mastering FE-IL who begins FE-TF starts effectively from scratch—and applies to the entire operating team.

Anatomic level is second. L5–S1 doses are 1.3–2× those at L4–5 and above, driven by iliac-crest obstruction and steep approach angles that require more oblique projections. No amount of operator experience eliminates these anatomic realities.

Patient body habitus exerts a large, under-appreciated effect: radiation output scales exponentially with tissue thickness. A patient with a body mass index (BMI) of 35 requires two to four times the tube output of a patient with a BMI of 25 for equivalent image quality, with correspondingly elevated surgeon scatter dose. Published ESS series rarely stratify dose by BMI.

C-arm geometry is largely operator-controllable: tube under the table, intensifier close to the patient, surgeon on the intensifier side during lateral shots. Compliance with these rules is inconsistent even among experienced operators.

Fluoroscopy mode and machine settings carry dose differentials of a full order of magnitude. Pulsed fluoroscopy at 7.5–15 pulses/s reduces dose by 50–75% versus continuous mode. Low-dose mode reduces further, with some image quality trade-off. Last-image-hold eliminates unnecessary beam-on time while the surgeon studies the image. Institutions reporting the lowest doses almost invariably use all of these as default; institutions reporting the highest doses typically do not.

Operator behaviour and workflow discipline—foot-pedal discipline, preplanned image sequences, step-back during acquisition, communication with the radiographer—account for a larger fraction of between-operator variance than machine settings alone and are the hardest determinant to modify through technology.

## 8. Dose Optimization Strategies

Available interventions range from cost-free behavioural changes to robotic systems costing over half a million US dollars. The evidence varies, and the patient-versus-surgeon distinction must be kept in mind—several widely promoted technologies reduce one party’s dose while increasing the other’s. Table 5 summarizes the principal interventions discussed in this section, indicating in each case whose dose is reduced, the typical magnitude of reduction, the approximate cost, and the strength of the supporting evidence.

### 8.1. Zero-Cost Technical Interventions

These require no additional equipment, only operator discipline, and are collectively among the highest-yield measures available. Collimation restricts the beam to the area of interest, reducing both patient entrance area and surgeon scatter. A prospective study during transforaminal PELD demonstrated a threefold reduction in surgeon chest-level dose from 0.108 to 0.039 mSv per case [11]. Pulsed fluoroscopy at 7.5–15 pulses/s reduces the dose by 50–75%. Low-dose mode reduces tube output further, with acceptable image quality for most ESS orientation tasks. Last-image-hold eliminates 20–40% of total beam-on time. Geometric discipline—tube under the table, intensifier close to the patient, surgeon on the intensifier side—can reduce the surgeon dose by 30–50%. Preoperative planning reduces confirmatory shots. Used together, these can plausibly reduce the total case dose by 50–70% without capital expenditure—a magnitude comparable to that claimed for many navigation or robotic systems.

### 8.2. Personal Protective Equipment

Standard 0.5 mm Pb lead aprons and thyroid collars reduce trunk dose by approximately 90%. Leaded eyewear (0.75 mm Pb) reduces eye-lens dose by 90% and is effectively mandatory for high-volume endoscopic practice under the revised ICRP limit [17]. Mobile lead shields provide an additional 60–90% reduction. Novel protective assemblies designed for ESS—exemplified by a prospective interventional study reporting surgeon dose reductions from 10.8 to 0.8 μSv at the chest and 10.2 to 0.7 μSv at the abdomen [20]—are underused in many practices. Ring and eye-lens-specific dosimeters are the essential feedback mechanism without which the above interventions cannot be verified.

### 8.3. Navigation, Robotics, and Next-Generation ESS Platforms

Preoperative CT-based navigation is best described as dose-shifting rather than dose-reducing: the burden transfers from the operating room to the preoperative scan (3–6 mSv for a lumbar CT), with intraoperative fluoroscopy reduced by 70–90% [7]. Intraoperative CBCT navigation (O-arm, Airo) effectively eliminates surgeon dose during acquisition but increases patient dose by 1–3 mSv per spin, and multi-spin protocols push single-level fusion above 10 mSv total [8]. The net effect is surgeon dose reduction with patient dose increase—the archetypal patient-versus-surgeon trade-off. Navigation accuracy (within 2 mm, 2°) is adequate for pedicle screws but may be marginal for endoscopic cannula docking. Robotic guidance paired with a single low-dose scan is the one mature technology that plausibly reduces both patient and surgeon dose simultaneously [23,24,25]. Cost (typically > USD 500,000), operative-time overhead, and a pronounced learning curve limit adoption to high-volume fusion practices.

A recent development specific to biportal ESS is fluoroscopy-based two-dimensional intraoperative computer navigation (2DNAV). A prospective comparative cohort by Park and colleagues in 20 biportal patients with 20 case-matched controls reported significant reductions in operative time, fluoroscopic images, and radiation exposure—mean radiation dose fell from 6.21 mGy with conventional C-arm to 0.77 mGy with 2DNAV [14]. This represents an intermediate category between pure fluoroscopy and full CBCT-based navigation, leveraging existing C-arm infrastructure without the capital cost of an O-arm, and is likely to be among the most accessible navigation modalities for mainstream biportal practice as evidence accumulates.

### 8.4. Emerging Technologies, Dosimetry Reporting, and Education

Augmented reality, electromagnetic tracking, and intraoperative ultrasound are at earlier stages of evidence [26]. Routine intraoperative dosimetry with real-time feedback reduces dose 15–30% through behavioural adjustment in other specialties and is under-deployed in spine surgery.

Standardized dosimetric reporting would improve the value of future ESS literature. We suggest that prospective ESS dosimetry studies report, at minimum: (i) DAP (Gy·cm^2^) and effective dose (mSv)—not fluoroscopy time alone; (ii) surgeon dosimeter readings at standardized anatomic sites (collar outside apron, waist beneath, finger ring, eye-lens-equivalent), with badge placement explicitly described; (iii) case-mix descriptors including operated level, patient BMI distribution, and the proportion of revision cases; (iv) machine settings (pulsed vs. continuous, frame rate, low-dose mode, collimation use); and (v) operator experience expressed as cumulative case number or years in independent practice. Aligning future ESS reporting with IAEA and ICRP frameworks would allow meaningful pooled analyses and meta-regression that the current literature does not support.

Educational implications deserve emphasis. Formal radiation-safety competency is embedded in interventional cardiology and interventional radiology training but is not yet a standard element of endoscopic spine fellowships. A practical curriculum component would include an introduction to the dosimetric concepts in Section 3, hands-on competency assessment in C-arm geometry and collimation, fitting and routine use of personal dosimeters, and structured review of each trainee’s accumulated case dose alongside the conventional procedural-volume log. Integrating radiation-safety competency into ESS training is among the lower-cost, higher-yield interventions available to the field. Institutional ALARA protocols and benchmarking through registries—neither currently standard in spine surgery—are the governance measures that allow these training-level changes to translate into measurable practice change.

### 8.5. Wearable Monitoring of Surgeon Ergonomics and Exposure

As noted in Section 6.2, occupational risk during ESS is multidimensional. Beyond ionizing radiation, the surgeon is exposed to ergonomic strain—sustained cervical flexion to view the endoscopic monitor, static upper-limb posture during prolonged cannula manipulation, and standing-only work that can extend across multi-level cases. Surveys of spine surgeons report a high prevalence of musculoskeletal complaints, and the ergonomic profile of endoscopic procedures has not been characterized to the same depth as the radiation profile. Crucially, posture and proximity also modulate radiation dose: a surgeon who leans toward the operative field for better visualization simultaneously increases scatter exposure, while one who maintains optimal distance and trunk inclination reduces both ergonomic load and dose.

A promising emerging approach is the integration of wearable inertial measurement units (IMUs) and motion-monitoring sensors with conventional dosimetry. Body-worn accelerometers, gyroscopes, and magnetometers can record surgeon posture, joint angles, trunk inclination, head position, and proximity to the radiation source in real time, complementing the cumulative-dose data provided by badge dosimeters. Wavelet-based and machine-learning signal-processing methods developed for human gait and movement analysis—exemplified by IMU-based feature-detection work in the wearable-robotics literature [27]—demonstrate that complex movement patterns can be reliably extracted from inertial sensor streams in biomedical settings, and the underlying methodology is directly transferable to the operating theatre environment. In principle, such systems could provide trainees with real-time feedback on posture and proximity during ESS, allow institutions to benchmark surgeon-level ergonomic and dose patterns, and support the design of evidence-based guidance on optimal positioning during fluoroscopy-guided procedures. Clinical implementation in spine surgery remains at an early stage, but the integration of wearable motion monitoring with established radiation-protection workflows represents a substantive interdisciplinary direction for future research and a logical extension of the surgeon-centred optimization agenda discussed throughout Section 8.

The intervention hierarchy carries a practical message. The largest available dose reductions in ESS come from the cheapest interventions when consistently applied. Collimation, pulsing, low-dose mode, geometric discipline, and leaded eyewear together deliver reductions comparable to those claimed for many navigation or robotic platforms, at a fraction of the cost. Capital-intensive technologies have a legitimate role—particularly for endoscopic fusion in high-volume practices—but they should supplement rather than substitute the zero-cost measures.

### 8.6. Clinical Recommendations Summary

Synthesizing the evidence reviewed above, the following practical recommendations may guide daily endoscopic spine practice. They are framed as guidance rather than mandates and should be adapted to the available institutional dosimetry data and the specific clinical context.

1. Choose the lower-radiation approach when clinically equivalent—for example, FE-interlaminar over FE-transforaminal at L5–S1, where the herniation pattern permits.
2. Set pulsed fluoroscopy, collimation, low-dose mode, and last-image-hold as the institutional default rather than leaving them to operator discretion.
3. Adopt 0.75 mm Pb leaded eyewear, a lead apron with thyroid collar, and a mobile lead shield for any high-volume fluoroscopy-dependent practice.
4. Maintain disciplined C-arm geometry: tube under the table, intensifier close to the patient, surgeon on the intensifier side during lateral projections.
5. Record patient effective dose in the operative report and wear properly positioned personal dosimeters (collar outside apron, ring, eye-lens-equivalent).
6. Use intraoperative CBCT and robotic guidance selectively where they reduce total combined patient + surgeon dose, particularly for endoscopic interbody fusion—not as defaults for routine decompression.
7. Integrate radiation-safety competency into ESS fellowship training, and review trainees’ accumulated case dose alongside procedural-volume logs.
8. Revisit institutional ALARA protocols periodically and benchmark against published dosimetric ranges; consider piloting wearable ergonomic and proximity monitoring where infrastructure allows.

## 9. Conclusions

Endoscopic spine surgery has matured rapidly, and the technical repertoire now spans full-endoscopic transforaminal and interlaminar procedures, biportal decompression, and endoscopic interbody fusion. Radiation exposure is one practical consideration that the community is thoughtfully working on as adoption continues to grow. The current evidence is reassuring in many respects: interlaminar full-endoscopic and most biportal decompression procedures sit comfortably within the range of tubular MIS, and the higher-dose scenarios—transforaminal approaches and endoscopic fusion—have well-characterized mitigation options. The goal is not to step away from fluoroscopy, which remains central to indirect visualization, but to use it thoughtfully.

Three practical takeaways may be useful in daily practice. First, discussing intraoperative radiation with patients during surgical consent is becoming the emerging standard, alongside the clear soft-tissue and recovery benefits that often make ESS the better overall choice. Second, patient and surgeon doses respond to different interventions, so it can be helpful to think of dose optimization as two complementary tracks. Intraoperative CBCT, for example, meaningfully reduces surgeon exposure while adding to patient dose, and the right balance depends on the case. Third, the interventions that often deliver the largest dose reductions are the most accessible—collimation, pulsed fluoroscopy, low-dose mode, sensible C-arm positioning, and leaded eyewear. Navigation and robotic platforms have a growing role, especially in endoscopic fusion, and complement these foundational measures rather than replace them.

Limitations of the underlying evidence should temper how these takeaways are applied. The reviewed literature consists predominantly of single-centre retrospective series and small prospective cohorts, with substantial heterogeneity in operator experience, patient case-mix, dosimetric metrics, and machine settings. Quantitative pooling across studies is therefore limited, and the dose ranges presented throughout this review should be interpreted as orders of magnitude rather than precise institution-level benchmarks. Large prospective, multicentre dosimetry registries—and dedicated spine-specific occupational cohorts—are not yet available, and several of the more confident claims that appear in the broader interventional radiology literature cannot yet be made with the same strength for endoscopic spine surgeons specifically. Readers are encouraged to weigh the recommendations here against their own institutional dosimetry data wherever available.

Looking ahead, routine dosimetry, continued refinement of training, and straightforward institutional ALARA protocols offer a practical path for the field. Standardized reporting of patient and surgeon doses in future ESS studies, alongside integration of radiation-safety competency into fellowship training, would meaningfully strengthen the evidence base over the next decade. Newer tools—including fluoroscopy-based 2D navigation for biportal ESS and, more speculatively, wearable inertial sensor and motion-monitoring platforms that integrate ergonomic and proximity data with conventional dosimetry—suggest that substantial improvements in surgeon-centred occupational risk can be achieved across multiple dimensions simultaneously. ESS continues to evolve, and incorporating simple dose-awareness and ergonomic-awareness habits into everyday practice is a natural next step in its development—one that benefits patients and surgeons alike.

## Figures and Tables

**Table 1:** Summary of key dosimetric studies in endoscopic and minimally invasive spine surgery referenced in this review.

First Author, Year	Study Type	Sample/Setting	Technique Studied	Key Dosimetric Endpoint
Rampersaud, 2000 [5]	In vitro/cadaveric	6 cadavers	Fluoroscopy-assisted pedicle screw	Surgeon hand/torso/neck dose; distance effect
Ahn, 2013 [6]	Prospective	30 cases, single centre	PELD	Surgeon dose during PELD; eye-lens, thyroid, hand
Villard, 2014 [7]	Prospective RCT	314 patients	Navigated vs. freehand pedicle screw	Surgeon and patient effective dose comparison
Iprenburg, 2016 [3]	Prospective	160 cases, single centre	Transforaminal full-endoscopic discectomy	Patient effective dose 1.5 (L4–5) → 2.1 mSv (L5–S1); learning-curve effect
Mendelsohn, 2016 [8]	Prospective comparative	73 patients	MIS fusion with CT-based navigation	Patient dose 1.09 mSv from O-arm; surgeon dose markedly reduced
Ishii, 2019 [9]	Prospective interventional	20 procedures	Full-endoscopic lumbar with novel shield	Surgeon chest dose 10.8 → 0.8 μSv; abdomen 10.2 → 0.7 μSv
Arif, 2021 [10]	Systematic review	15 studies	MIS spine—surgeon dose	Pooled surgeon dose ranges, intervention efficacy
Erken, 2022 [11]	Prospective comparative	40 cases	Endoscopic spine surgery	Collimation: surgeon dose 0.108 → 0.039 mSv (~64% ↓)
Jiang, 2022 [12]	Retrospective comparative	80 patients (40/40)	UBE vs. PELD	Fluoroscopy time and frequency comparison
He, 2023 [13]	Systematic review/meta-analysis	12 studies, ~1200 patients	UBE vs. PELD	Pooled fluoroscopy time comparison favouring UBE
Park, 2025 [14]	Prospective comparative cohort	40 patients (20/20)	Biportal ESS with 2DNAV vs. C-arm	Mean radiation 6.21 → 0.77 mGy (~85% ↓); operative time reduced
Helton, 2025 [4]	Systematic multi-specialty review	Cross-specialty	Occupational fluoroscopy across surgical disciplines	Cross-specialty surgeon-dose ranges and risk factors

PELD, percutaneous endoscopic lumbar discectomy; UBE, unilateral biportal endoscopy; MIS, minimally invasive surgery; 2DNAV, fluoroscopy-based two-dimensional intraoperative navigation; mSv, millisievert; μSv, microsievert; mGy, milligray. Studies were selected to illustrate (a) prospective dosimetric methodology, (b) the principal head-to-head comparisons informing Section 4 and Section 5, and (c) the evidence base for the optimization strategies in Section 8. →, a change from the first value to the second. ↓, a dose decrease.

**Table 2:** ICRP occupational dose limits relevant to endoscopic spine surgery.

Exposure Site	Annual Limit	Notes
Whole body (effective dose)	20 mSv/year, 5-year average	Not to exceed 50 mSv in any single year
Eye lens	20 mSv/year, 5-year average	Reduced from 150 mSv in 2011
Hands, feet, skin	500 mSv/year	Ring/extremity dosimeter
Declared pregnancy (conceptus)	1 mSv over remainder of gestation	Triggers mandatory work-practice review
Patient	No fixed limit	Governed by ALARA and justification

ICRP, International Commission on Radiological Protection; ALARA, as-low-as-reasonably-achievable; mSv, millisievert. Adapted from ICRP Publications 103 and 118 [5,6].

**Table 3:** Characteristic radiation signatures of the main endoscopic spine techniques.

Technique	Fluoroscopy Time (Single Level)	Patient Effective Dose	Principal Dose Driver
FE transforaminal (FE-TF)	30–90 s	0.6–4 mSv	Cannula docking, foraminoplasty, L5–S1 angulation
FE interlaminar (FE-IL)	5–15 s	<1 mSv	Level localization only
UBE/BESS	10–25 s	<1–1.5 mSv	Portal placement; rises with contralateral work
Endoscopic interbody fusion	60–180 s	3–8 mSv	Pedicle-screw placement, cage insertion

FE, full-endoscopic; FE-TF, full-endoscopic transforaminal; FE-IL, full-endoscopic interlaminar; UBE, unilateral biportal endoscopy; BESS, biportal endoscopic spinal surgery; mSv, millisievert. Ranges pooled from series 2010–2025. Where original studies reported fluoroscopy time only, dose ranges are approximated and should be interpreted as indicative rather than precise.

**Table 4:** Patient effective dose by procedure and approach.

Procedure	Approach	Patient Effective Dose (mSv)	Principal Driver
Lumbar discectomy	Open microdiscectomy	<0.1	Minimal fluoroscopy
	Tubular MIS	0.2–0.5	Tube docking
	FE interlaminar (L5–S1)	<1	Level localization
	FE transforaminal	1.5–2.1 (experienced)	Cannula docking, foraminoplasty
Lumbar interbody fusion	Open TLIF/PLIF	1–3	Direct anatomy
	MIS-TLIF (fluoroscopic)	4–10	Percutaneous pedicle screws
	Endo-TLIF/UBE-TLIF	3–8	Decompression + screws
	Any MIS fusion + O-arm	+1–3 per spin	Intraoperative CBCT

FE, full-endoscopic; MIS, minimally invasive surgery; TLIF, transforaminal lumbar interbody fusion; PLIF, posterior lumbar interbody fusion; UBE, unilateral biportal endoscopy; CBCT, cone-beam computed tomography; mSv, millisievert. Values approximate the central tendency across published series and should not be taken as institution-level benchmarks.

**Table 5:** Dose-optimization interventions in endoscopic spine surgery.

Intervention	Whose Dose Reduced	Typical Reduction	Cost	Evidence
Collimation	Patient and surgeon	50–70% surgeon	Zero	Strong
Pulsed fluoroscopy	Patient and surgeon	50–75%	Zero	Strong
Low-dose mode	Patient and surgeon	30–50%	Zero	Moderate
Last-image-hold	Patient and surgeon	20–40% beam time	Zero	Moderate
Geometric discipline	Surgeon	30–50%	Zero	Moderate
Lead apron + thyroid collar	Surgeon	90% trunk	Low	Strong
Leaded eyewear	Surgeon (eye lens)	90%	Low	Strong
Mobile lead shield	Surgeon	60–90% additional	Low–mod	Strong
Preoperative CT navigation	Surgeon; patient shifted	70–90% fluoroscopy	Moderate	Moderate
CBCT navigation	Surgeon; patient ↑	Surgeon 70–90%; patient +1–3 mSv/spin	High	Moderate
2DNAV (biportal ESS)	Patient and surgeon	~85% dose (6.21 → 0.77 mGy)	Moderate	Early (prospective)
Robotic guidance	Both	Surgeon 70–90%; patient ↓ vs. CBCT	Very high	Moderate
Dosimetry with feedback	Surgeon	15–30% additional	Low	Moderate
Training + ALARA protocols	Both	20–50%	Low	Indirect

CT, computed tomography; CBCT, cone-beam computed tomography; 2DNAV, fluoroscopy-based two-dimensional intraoperative navigation; ALARA, as-low-as-reasonably-achievable; ESS, endoscopic spine surgery; mSv, millisievert; mGy, milligray; Pb, lead. “Evidence” reflects strength relative to typical ESS dosimetry literature, not formal GRADE assessment. ↑, a dose increase; →, a change from the first value to the second; ↓, a dose decrease.

## Data Availability

No new data were created or analyzed in this study. Data sharing is not applicable to this article.

## Abbreviations

The following abbreviations are used in this manuscript:

2DNAV	Fluoroscopy-based two-dimensional intraoperative navigation
ALARA	As-low-as-reasonably-achievable
BESS	Biportal endoscopic spinal surgery
BMI	Body mass index
CBCT	Cone-beam computed tomography
CT	Computed tomography
DAP	Dose–area product
Endo-TLIF	Full-endoscopic transforaminal lumbar interbody fusion
ESS	Endoscopic spine surgery
FE	Full-endoscopic
FE-IL	Full-endoscopic interlaminar approach
FE-TF	Full-endoscopic transforaminal approach
IAEA	International Atomic Energy Agency
ICRP	International Commission on Radiological Protection
IMU	Inertial measurement unit
MIS	Minimally invasive surgery
MIS-TLIF	Minimally invasive transforaminal lumbar interbody fusion
mGy	Milligray
mSv	Millisievert
PELD	Percutaneous endoscopic lumbar discectomy
PLIF	Posterior lumbar interbody fusion
PPE	Personal protective equipment
SANRA	Scale for the Assessment of Narrative Review Articles
TLIF	Transforaminal lumbar interbody fusion
UBE	Unilateral biportal endoscopy
UBE-TLIF	Biportal endoscopic transforaminal lumbar interbody fusion
